# The intersection between toxicology and aging research: A toxic aging coin perspective

**DOI:** 10.3389/fragi.2022.1014675

**Published:** 2022-09-21

**Authors:** John P. Wise Jr

**Affiliations:** NeuroWise Laboratory of Environmental Neurotoxicology, Pediatrics Research Institute, Department of Pediatrics, University of Louisville, Louisville, KY, United States

**Keywords:** gerontogen, aging, toxicology, neuroinflamation, blood brain barrier (BBB), cellular senescence

## Abstract

We are imminently faced with the challenges of an increasingly aging population and longer lifespans due to improved health care. Concomitantly, we are faced with ubiquitous environmental pollution linked with various health effects and age-related diseases which contribute to increased morbidity with age. Geriatric populations are rarely considered in the development of environmental regulations or in toxicology research. Today, life expectancy is often into one’s 80s or beyond, which means multiple decades living as a geriatric individual. Hence, adverse health effects and late-onset diseases might be due to environmental exposures as a geriatric, and we currently have no way of knowing. Considering aging from a different perspective, the term “gerontogen” was coined in 1987 to describe chemicals that accelerate biological aging but has largely been left out of toxicology research. Thus, we are challenged with a two-faced problem that we can describe as a “toxic aging coin”; on one side we consider how age affects the toxic outcome of chemicals, whereas on the other side we consider how chemicals accelerate aging (i.e. how chemicals act as gerontogens). Conveniently, both sides of this coin can be tackled with a single animal study that considers multiple age groups and assesses basic toxicology of the chemical(s) tested and aging-focused endpoints. Here, I introduce the concept of this toxic aging coin and some key considerations for how it applies to toxicology research. My discussion of this concept will focus on the brain, my area of expertise, but could be translated to any organ system.

## 1 Introduction

We are entering a new international paradigm where environmental pollution is ubiquitous and coinciding with an unprecedented aging population. Today people are living 50 years longer than in 1950, and ∼10% of the global population is geriatric (aged 65+). Further, the United Nations projects by 2030, 20% of the population in developed nations will be geriatric, with the global population reaching this proportion by 2075 (United Nations et al., 2019; Gu et al., 2021). This dynamic presents new challenges to our health infrastructure as age-related diseases and comorbidities become increasingly prevalent in our populations. For many of these age-related diseases, environmental pollutants are significant contributors to their etiology and we will continue to see a rise in their prevalence unless we are able to reduce or remediate environmental pollution. Compounding this issue, we now recognize many of these pollutants may act as gerontogens—chemicals that induce or accelerate the biological aging process (Martin, 1987). Hence, we are challenged with a two-faced issue that we can refer to as a “toxic aging coin”: on the heads side we consider how age impacts the toxic outcome of a chemical, whereas on the tails side we consider how chemicals act as gerontogens. Here, I will discuss how this toxic aging coin applies to brain health, though this principle could be applied to any organ system. Importantly, our ability to investigate this toxic aging coin is enhanced by several key papers from the last decade describing the hallmarks of aging, hallmarks of brain aging (which are slightly different), and reviews describing the toxicology of aging (López-Otín et al., 2013; Sorrentino et al., 2014; Mattson and Arumugam, 2018; Rackova et al., 2021). This article will focus on the aspects of aging and gerontogens that are most impactful, and provide references to reviews that discuss relevant topics in further detail.

## 2 Heads: Age impacts neurotoxic effects of chemicals

The *heads* side of this toxic aging coin considers how age can change the neurotoxic effect of chemicals. From decades of lifespan research, we recognize the biology of our brains are distinct across ages which we can summarize simply as young, adult, or geriatric; a young brain is developing, an adult brain is mature, and a geriatric brain is degenerating. With these three distinctions, we can begin to conceptualize how age can alter the ways chemicals interact with our brains across our lifespan. Importantly, geriatric populations have largely been left out of the framework of environmental toxicology and safety regulations, leaving a huge gap in our understanding the toxicology of aging. In this Section 1 will discuss the heads side of the toxic aging coin, and how we as toxicologists should consider all age groups in our research—young, adult, and geriatric.

Neurodevelopmental exposures may occur pre- or postnatally, and the timing of exposure can have critically different impacts. The development of the nervous system begins relatively late during embryogenesis, and many of the mechanisms driving neural development are conserved across species. After the three main cell layers are formed (endoderm, mesoderm, ectoderm), the nervous system develops from the ectoderm during the gastrula stage. Neurodevelopment proceeds through tightly regulated signaling molecules to direct migration of neurons and axons to their terminal destinations, which may be affected by toxicants (Connors et al., 2008; Batool et al., 2019). Importantly, neurodevelopment features an initial overproduction of neurons followed by a period of cell death; both processes can be affected by toxicants. Hence, we can see environmental chemicals may disrupt neurodevelopment at multiple stages with drastically different outcomes (Rodier, 1995; Rice and Barone, 2000; Heyer and Meredith, 2017). The most studied outcomes of neurodevelopmental toxicology are neurodevelopmental disorders (e.g. autism spectrum disorder, attention deficit/hyperactivity disorder) and late-life neurodegenerative diseases following the Developmental Origin of Health and Disease (DOHaD) hypothesis (Schmid and Rotenberg, 2005; Wadhwa et al., 2009; Gluckman et al., 2010; Patisaul, 2017; Tran and Miyake, 2017; van de Bor et al., 2019). After birth, our brains continue to grow and change for much of our lives; typically, our brains are ∼80% of the maximum size by age 3, with the maximum gray matter, subcortical gray, and white matter volumes reached by age 6, 14, and 30, respectively (Bethlehem et al., 2022). After maximum brain volume is reached, regional declines are observed with age and each region exhibits a different rate of volume decline (Ziegler et al., 2012; Tamnes et al., 2013; Fuhrmann et al., 2022). After age 40 the volume of brain ventricles begins to rapidly increase, and after age 50 the white matter volume rapidly declines with age (Bethlehem et al., 2022).

### 2.1 The neurovascular unit and blood-brain barrier across ages

A critical component for distinguishing how environmental chemicals may impact brain health across ages is the blood brain barrier (BBB), a functional barrier formed by cells within the neurovascular unit (NVU). The NVU is made up of cerebrovascular endothelial cells, pericytes, and astrocytic endfeet, with neurons and microglia closely associated with the periphery to stimulate or respond to NVU functions as needed (Muoio et al., 2014; Liu et al., 2020). The BBB is formed by cerebrovascular endothelial cells at the start of angiogenesis during neonatal development (embryonic day 11 in rats), and express proteins that provide roles in structural, transport, metabolic, and adhesion properties distinct from endothelial cells in other tissues (Blanchette and Daneman, 2015). These early blood vessels initially exhibit leaky properties such as high rates of transcytosis and leukocyte adhesion molecules (LAMs). Leakiness decreases within the first few days following angiogenesis by endothelial cells, increasing the complexity of barrier proteins (e.g. TJ-1, ZO-1, occludin, claudins) and recruiting pericytes, which suppress endothelial expression of LAMs and transcytosis (Armulik et al., 2010; Daneman et al., 2010). Astrocyte endfeet cover almost the entire surface of microcapillaries in the brain, but astrogliogenesis begins after birth. Importantly, the interaction between astrocytic endfeet and cerebrovascular endothelial cells determines the final maturation of the NVU (Wolburg et al., 2009; Gilbert et al., 2019). Hence, there is a post-natal period where the NVU is not completely developed and infant brains are at higher risk for chemical insults. Upon maturation, the NVU/BBB serve to protect the brain parenchyma from peripheral blood components through barriers and efflux channels, while also providing nutrients through specific transport proteins (Daneman and Prat, 2015).

It is unclear when (or if there is) an age threshold that defines when a mature NVU/BBB shifts to a degenerative state. During aging it is clear there are several phenotypic changes that occur that contribute to leakiness in the degenerating cerebrovasculature, including: focal necrosis of endothelium, decreased endothelial mitochondrial density, increased pinocytic vesicles, loss of tight junction proteins, loss of astrocytic endfeet, stiffening of vessel walls, decreased microvascular density; and smaller capillary lumen size with greater tortuosity (Zeevi et al., 2010; Marques et al., 2013). These degenerative changes to the NVU/BBB are believed to contribute to the etiology of age-associated neurodegenerative diseases (e.g. Alzheimer’s disease, Parkinson’s disease, amyotrophic lateral sclerosis), age-associated susceptibility to stroke, white matter disease, delirium, and other age-associated neurological conditions. In sum, we exhibit a weaker BBB for a relatively short window (months) early in our development and for a much longer time (years to decades) toward the end of our lifespan. During these periods our brains are susceptible to insult by a wider variety of chemical insults.

### 2.2 Neuroinflammation across lifespan

Neuroinflammation is a defensive response to injury or infection to protect the brain and promote repair, primarily directed by microglia and astrocytes. Imbalance of neuroinflammation activity early in life can contribute to development of neurodevelopmental disorders, whereas later in life it can contribute to development of neurodegenerative diseases (Molofsky et al., 2012; Chen et al., 2016; Petrelli et al., 2016; Kim et al., 2020). Clinically, exaggerated neuroinflammation contributes to depression, cognitive impairment, memory loss, delirium, epilepsy, sickness behavior, and neurodegenerative diseases (Lyman et al., 2014). Microglia play the most significant role in neuroinflammation, shifting from a ramified, resting state to an activated phagocytic state in response to inflammatory stimuli, and can remain activated for long periods of time. While activated, microglia release pro-inflammatory cytokines (e.g. IL-6, TNF-α, IL-1β) and neurotoxic molecules (e.g. nitric oxide, NO) that contribute to impaired synaptic plasticity, loss of synapses, neuronal apoptosis, and long-term neurodegeneration (Lyman et al., 2014). Microglia become primed due to peripheral inflammation, toxicants, or normal aging, which results in an exaggerated response to secondary stimuli (e.g. infection, toxicants, stress) (Lyman et al., 2014; Barrientos et al., 2015). While the number of microglia does not change with age, the expression of activation markers including major histocompatibility complex II (MHC II), CD11b, and Iba-1 increase on a per cell basis and secrete higher amounts of pro-inflammatory cytokines (Lyman et al., 2014). Normally, neuroinflammatory changes in microglia are transient and return to a resting, ramified state after the insult is cleared. Aging may present a context where microglia activation is not resolved and persists, resulting in a chronic state of primed microglia and exaggerated responses to noxious stimuli. Microglia can also recruit astrocytes to participate in neuroinflammation (Godbout and Johnson, 2009). When activated, astrocytes increase expression of glial fibrillary protein (GFAP). Depending on context and timing, this activation may contribute to immunosuppression and tissue repair, or it may exacerbate inflammatory reactions and tissue damage (Colombo and Farina, 2016). Overall, astrocytes display a more inflammatory phenotype with aging. Importantly, pro-inflammatory cytokines from microglia and astrocytes impair the integrity of the BBB, altering the resistance of tight junctions, increasing permeability, and enabling peripheral leukocytes to enter the brain (Terrando et al., 2011).

## 3 Tails: Chemicals act as gerontogens to accelerate biological aging

The *tails* side of this toxic aging coin considers how chemicals can accelerate biological aging. The role of genetics account for 10–25% of human lifespan, thus non-genetic, epigenetic, and environmental factors must play a significant role in human aging (Ruby et al., 2018; van den Berg et al., 2019; Bin-Jumah et al., 2022). The term “gerontogen” was first coined in 1987 by Dr. George Martin to describe the effect of chemicals inducing or accelerating biological aging processes in tissues and cells (Martin, 1987). At the time of this publication, there was a detrimental lack of reliable aging biomarkers to support thorough investigation of gerontogens causing aging phenotypes. Hence, a proper investigation of aging toxicology had to wait. Since then, there has been a robust research effort to understand the genetics behind biological aging, and a push to better understand underlying gene-environment interactions (Martin, 1997; Rodríguez-Rodero et al., 2011; Moskalev et al., 2017; Singh et al., 2019). Yet the field of toxicology has lagged in recognizing the significance of aging as a toxicological phenotype, with relatively few studies investigating gerontogenic effects of chemicals. Today, however, the stage has been set and toxicology needs to step into the limelight. Nine hallmarks of aging were identified and described in 2013, and a distinct set of hallmarks for brain aging were described in 2018, that now enable proper mechanistic investigation of gerontogens (López-Otín et al., 2013; Mattson and Arumugam, 2018). Further, several animal models have been developed for rigorous aging studies ranging from *C. elegans* to fish to rodents (Holtze et al., 2021). With the advent of these aging hallmarks and research models, toxicologists now have the tools to mechanistically investigate gerontogens as a class of chemicals. In this Section 1 will discuss the tails side of this toxic aging coin perspective, and how we as toxicologists can capitalize on the available resources to investigate how chemicals may act as gerontogens, and I will note key review articles where further discussion may be warranted.

An aging brain manifests as deficits in learning and memory, attention, sensory perception, motor coordination, decision-making speed, and cognitive function (Yankner et al., 2009; Machado, 2020). Many of these are observed in well-known age-related neurodegenerative diseases (e.g. Alzheimer’s disease, Parkinson’s disease, amyotrophic lateral sclerosis, stroke). With our rapidly growing geriatric population we can expect to see a similar significant increase in these age-related neurodegenerative diseases. It is projected we will see the number of people diagnosed with Alzheimer’s disease more than double in the next 30 years from ∼ 5 million today to more than 12 million (Alzheimer’s Association, 2016). Similar to aging, these neurodegenerative diseases are believed to have a significant environmental influence, with only 5–10% of cases linked to genetic mutations driving their etiology (Bertram and Tanzi, 2005; Philstrøm et al., 2018). Faced with this societal challenge, it is imperative we understand how environmental chemicals contribute to brain aging and age-related neurodegenerative diseases and find ways to prevent or reduce exposures to such chemicals (Cannon and Greenamyre, 2011; Cicero et al., 2017).

The defined hallmarks of brain aging and that of peripheral tissues are slightly different. While there is quite a bit of overlap, two hallmarks of aging in peripheral tissues are still emerging in the context of brain aging, but are likely particularly relevant for the brain’s glial cells; cellular senescence and telomere attrition. Given that glial cells make up ∼50% of brain cells and displayed the strongest effect of differential gene expression in aging, it is probable these glial cells will have stronger deterministic effects in aging than neurons (Soreq et al., 2017).

### 3.1 Contributions of cellular senescence to brain aging

Perhaps the most important hallmark for assessing gerontogenic effects of chemicals is cellular senescence, defined as a state of permanent cell cycle arrest, as it is a terminal cellular phenotype that contributes to tissue/organ aging (López-Otín et al., 2013; Muñoz-Espín and Serrano, 2014; Hernandez-Segura et al., 2018; González-Gualda et al., 2021). This view is supported by various studies using senolytics to selectively induce apoptosis in senescent cells and showing improvement in age-related pathology (Baker and Petersen, 2019; Zhang et al., 2019; Ogrodnik et al., 2021; Gonzales et al., 2022; Kim et al., 2022). From a reductionist point of view; abundance of senescent cells marks aging organs and, ultimately, aging bodies (Campisi, 2001). Senescent cells become more abundant with normal aging, are elevated in neurodegenerative diseases, and age-related symptoms can be alleviated with their selective removal by senolytic or senomorphic drugs (Kritsilis et al., 2018; Martínez-Cué and Rueda, 2020; Sikora et al., 2021; Kim et al., 2022). Thus, this hallmark is likely the most important for determining if a toxicant is a gerontogen.

Cellular senescence is characterized by an enlarged, flattened morphology, altered gene expression (e.g. increased *p21, p16* ^*INK4a*^ *, C-fos, PCNA*), increased senescence associated-β-galactosidase (SA-β-gal) staining, altered mitochondrial function and morphology, altered cellular metabolism, and expression of the senescence associated secretory phenotype (SASP) (Hernandez-Segura et al., 2018; Martínez-Cué and Rueda, 2020). Several molecular pathways contribute to cells shifting to a senescent state, though many of the mechanisms for these pathways have yet to be elucidated. These pathways include, but are not limited to: DNA damage response, oxidative stress, telomere shortening, mitochondrial dysfunction, loss of proteostasis, and endoplasmic reticulum stress (Ben-Porath and Weinberg, 2005; Hernandez-Segura et al., 2018; Wei and Ji, 2018; Di Micco et al., 2020).

The impact of cellular senescence on brain health depends on the type of brain cell, but likely senescence of cells making up the NVU will have the most significant role in brain aging. A dysfunctional or impaired NVU will result in transient capillary occlusion, BBB leakage of blood proteins, pro-inflammatory SASP expression, neurovascular uncoupling, and impaired blood/nutrient supply to the brain, collectively promoting neurodegeneration (Cortes-Canteli et al., 2015; Bennett et al., 2018; Petersen et al., 2018; Bryant et al., 2020). The vast majority of chemical exposures to the brain will be delivered by the blood; hence the first brain cells at risk of gerontogens will be brain endothelial cells in the cerebrovasculature (Graves and Baker, 2020). Senescent endothelial cells shift their phenotype to express more surface leukocyte adhesion molecules (VCAM-1, ICAM-2, MAdCAM-1) and generate a proinflammatory environment to recruit peripheral immune cells to the senescent tissue, contributing to transient capillary occlusion (Gorgoulis et al., 2005; Lasry and Ben-Neriah, 2015; Bryant et al., 2020). BBB leakage, evidenced by increased extravasation of IgG and decreased expression of tight junction proteins, is another critical effect of aging on the NVU which likely precedes onset of neurodegenerative diseases (Pelegri et al., 2007; Bake et al., 2009; Yamazaki et al., 2016). Astrocytes can become pathogenic when senescent (Cohen and Torres, 2019). Evidence shows increased proportion of senescent astrocytes in the frontal cortex of AD patients and senescent astrocytes can induce neuronal apoptosis or synaptic dysfunction (Pertusa et al., 2007; Bhat et al., 2012; Miranda et al., 2012). Importantly, one study demonstrated the selective clearance of senescent astrocytes prevented tau deposition and degeneration of hippocampal and cortical neurons in an AD mouse model (Bussian et al., 2018). Similarly, microglia can become senescent and contribute to neuroinflammation and neurodegeneration, though these glia have not been as extensively studied and it is unclear if there is a distinction necessary for senescent vs. dystrophic microglia (Angelova and Brown, 2019). Aging microglia were initially identified by morphology in brains of aged individuals, and have subsequently been shown to exhibit impaired motility, iron accumulation, DNA damage accumulation, telomere shortening, and SASP-like changes in cytokine production (Streit, 2004; Sierra et al., 2007; Hayashi et al., 2008; von Bernhardi et al., 2015; Flanary and Streit, 2004; Orre et al., 2014; Simmons et al., 2007). Given the abundance of evidence for cellular senescence in brain aging and neurodegenerative diseases, and the growing evidence for senolytics as a preventative or therapeutic strategy for these conditions, it should be added to the hallmarks of brain aging. Further, we need more toxicology-based research to identify which chemicals contribute to cellular senescence and what senescent mechanisms or pathways they induce.

## 4 Concluding remarks

Considering the new societal shift we are imminently facing with a large geriatric population, we need a similar shift in toxicology research to investigate how chemicals interact with this toxic aging coin. Both sides of the toxic aging coin will be imminently relevant and important to the health and success of our societies. We need more research groups investigating this intersection of toxicology and aging for all major organs. If all three age groups (young, middle-aged, geriatric) are included in a toxicology study, one can readily consider both sides of the toxic aging coin within the same study; on one side, this study design enables assessing how toxic effects are different across the age groups, and on the other side, it enables assessing how toxicants affect aging hallmarks.

## Data Availability

The original contributions presented in the study are included in the article/Supplementary Material; further inquiries can be directed to the corresponding author.
